# "Raising a Ghost It Were Better to Lay"

**Published:** 1917-10-27

**Authors:** C. O. Hawthorne


### THE EDITOR'S LETTER-BOX.

### "Raising a Ghost it Were Better to Lay."

*To the Editor of* The Hospital.

Sir,—I am obliged for the note printed after my letter in your issue of the 20th inst. It is gratifying to know that the writer agrees with me in affirming that payment for professional services rendered to State pensioners on the one hand, and payment for similar services rendered to the sick poor on the other, are two different issues. In the concluding sentences of his original article he had spoken of the "whole question" and of the necessity of raising "the question . . . completely," with, at least so it seems to me, the inevitable implication that the two issues which he now allows to be distinct were necessarily involved in one and the same debate. What other interpretation can possibly be attached to his contention that "if it [the question] is to be raised now, it should be raised completely"? His argument plainly is that the persons he criticises have raised the question in a partial or incomplete form, while he himself "prefers" to deal with the "whole question." There is no suggestion here of that separation of the two issues which is now so manifest to him. For any misunderstanding on this point he must therefore blame the careless quality of his phrases.

For a second misunderstanding my own ignorance may perhaps be responsible. The suggestion of the original article was that the B.M.A., while raising the question of payment for professional aid given to State pensioners, intended to "side-track" the payment of such payments for aid given to the sick poor. Innocently I concluded that "to side-track" meant to push aside in order to conceal or to hide from view the topic thus treated. I now learn this to be a mistake. To "side-track," it seems, means "a desire to proceed" to the discussion of the "side-tracked" proposition. At least this appears to be the only available explanation of the fact that while in the original article the B.M.A. is accused of having "side-tracked" the question of payments on behalf of the sick poor, the comment made upon my letter accuses the Association of a "desire to proceed" to the discussion of this question, and suggests that its "recent policy" is framed with this intention. I must penitently allow that it never occurred to me to suppose that to "side-track" a proposition meant a "desire" to discuss it as the next item of business.

As I have not the slightest title or warrant to speak for the B.M.A. I cannot pretend to say what "preferences" or "desires" it may cultivate in the future. But I do know that in the past it has repeatedly laid down the policy that services rendered to the State, or to those for whom the State is responsible, ought to be met by State payments, and I cannot doubt that it is ready to apply this doctrine to all cases which such doctrine covers. It is true that in voluntary hospitals and in V.A.D. hospitals a number of individual physicians and surgeons, looking upon the position as a temporary and exceptional one, have preferred to serve wounded soldiers without payment. But such a line of action in no way commits the B.M.A., which, indeed, in its recent representative meeting, strongly supported the claim for payment advanced by some of the V.A.D. practitioners. So far as I know no similar claim has been presented by the staffs of voluntary hospitals engaged in the treatment of sick and wounded soldiers. But were such a claim advanced there cannot be the slightest doubt that the Association would support it. It is in the contrast between these two positions that is to be found an explanation of the proceeding in which the writer detects evidence of a desire on the part of a section of the medical profession to "milk" a public authority, "to make hay while the sun shines," and generally to cultivate an ambition which it would be an "exaggeration" to describe as "grab." He does not again refer to these literary gems, but we must not suffer his modesty to weaken our memory of what we owe to his pen.—I am, Sir, yours faithfully, C. O. Hawthorne.

October 22, 1917.

